# Spatial Factors Outperform Local Environmental and Geo-Climatic Variables in Structuring Multiple Facets of Stream Macroinvertebrates’ *β*-Diversity

**DOI:** 10.3390/ani12192648

**Published:** 2022-10-02

**Authors:** Naicheng Wu, Guohao Liu, Min Zhang, Yixia Wang, Wenqi Peng, Xiaodong Qu

**Affiliations:** 1 Department of Geography and Spatial Information Techniques, Ningbo University, Ningbo 315211, China or; 2 State Key Laboratory of Simulation and Regulation of Water Cycle in River Basin, China Institute of Water Resources and Hydropower Research, Beijing 100038, China; 3 Department of Water Ecology and Environment, China Institute of Water Resources and Hydropower Research, Beijing 100038, China

**Keywords:** functional, Hun-Tai river, nestedness, phylogenetic, taxonomic, turnover, *β*-diversity partitioning

## Abstract

**Simple Summary:**

One of the key targets of community ecology and biogeography concerns revealing the variability and underlying drivers of biodiversity. Most current studies understand biodiversity based on taxonomic information alone. Our study was based on macroinvertebrates from 179 stream sampling sites in the Hun-Tai River Basin in Northeastern China. The correlation of different facets of *β*-diversity was compared while revealing the relative contribution of multiple abiotic factors (i.e., local environmental, geo-climatic, and spatial factors) to shaping *β*-diversity based on taxonomic, functional, and phylogenetic information. The results showed that functional *β*-diversity provides important complementary information to taxonomic and phylogenetic *β*-diversity. Moreover, spatial factors outperform local environmental and geo-climatic variables in structuring multiple facets of stream macroinvertebrates’ *β*-diversity. Our study provides guidance for future conservation studies of watershed biodiversity, as well as implications for future studies of *β*-diversity.

**Abstract:**

One of the key targets of community ecology and biogeography concerns revealing the variability and underlying drivers of biodiversity. Most current studies understand biodiversity based on taxonomic information alone, but few studies have shown the relative contributions of multiple abiotic factors in shaping biodiversity based on taxonomic, functional, and phylogenetic information. We collected 179 samples of macroinvertebrates in the Hun-Tai River Basin. We validated the complementarity between the three facets and components of *β*-diversity using the Mantel test. Distance-based redundancy analysis and variance partitioning were applied to explore the comparative importance of local environmental, geo-climatic, and spatial factors on each facet and component of *β*-diversity. Our study found that taxonomic and phylogenetic total *β*-diversity was mainly forced by turnover, while functional total *β*-diversity was largely contributed by nestedness. There is a strong correlation between taxonomic and phylogenetic *β*-diversity. However, the correlations of functional with both taxonomic and phylogenetic *β*-diversity were relatively weak. The findings of variation partitioning suggested that distinct facets and components of macroinvertebrates’ *β*-diversity were impacted by abiotic factors to varying degrees. The contribution of spatial factors was greater than that of the local environment and geo-climatic factors for taxonomic, functional, and phylogenetic *β*-diversity. Thus, studying different facets and components of *β*-diversity allows a clearer comprehension of the influence of abiotic factors on diversity patterns. Therefore, future research should investigate patterns and mechanisms of *β*-diversity from taxonomic, functional, and phylogenetic perspectives.

## 1. Introduction

Uncovering the comparative significance of the underlying drivers of biodiversity patterns has long been an important research subject in ecology and biogeography [1,2]. In the past, taxonomy-based studies have been conducted to reveal the community composition of organisms in specific areas [3,4,5]. However, this taxonomic approach failed to recognize that functional and phylogenetic variations exist between species. Recently, community ecologists have become aware of the need to start not just with taxonomic level information, but with functional and phylogenetic perspectives, so as to increase our comprehension of the relative contributions of the various factors that shape the patterns of biodiversity [6,7,8]. In addition, considering community ecology in conjunction with functional or phylogenetic perspectives enables us to further explore the ecological and evolutionary mechanisms that influence the compositional structure of communities; similar studies have covered several different aquatic species, including zooplankton, fish, and benthic algae [9,10,11]. 

Functional traits have been applied in biogeography over the past decades and have proven to be a favorable method for studying the relationship between environmental factors and biodiversity [8,12]. The reason is that traits can reflect the effects of the environment on the species (i.e., ecological, biological, and morphological-behavioral features), and functional diversity is consequently considered to be more strongly linked to the environment than taxonomic diversity [13,14,15]. Considering the influence of various drivers (e.g., spatial factors, local environment, and geo-climate) on functional traits can provide clearer insight into the processes of community construction [16,17,18].

Additionally, phylogenetic diversity has received increasing attention in current biodiversity research. Phylogenetic diversity captures the evolutionary history of species within a community and also demonstrates information about species in the context of differentiation (i.e., relationships among species) and richness (i.e., the amount of cumulative evolutionary history) [19,20]. Since the identity of species in a community depends heavily upon evolutionary history, phylogenetic characteristics determine species’ ability to produce new resolutions in the face of future changes in environmental contexts [21,22]. However, few studies have so far simultaneously investigated the mechanisms and drivers of diversity based on three facets: taxonomic, functional, and phylogenetic.

*β*-diversity consists of two components: turnover and nestedness [23]. Nestedness means that a species-poor area is a subset of a species-rich site, while turnover means species replacement between sites [24,25]. In addition, *β*-diversity can be divided into taxonomic, functional, and phylogenetic *β*-diversity [26]. While recognizing that different facets of biodiversity can be complementary and uncover information about the community compositions [27], studies analyzing the ecological factors of taxonomic, functional, and phylogenetic diversity of macroinvertebrates in freshwater ecosystems are still scarce [8,28].

This study explores the comparative significance of various factors (i.e., spatial factors, local environmental, and geo-climatic variables) in shaping the taxonomic, functional, and phylogenetic macroinvertebrates’ *β*-diversity in the Hun-Tai River Basin. In our study, we tried to answer three major questions: (i) What is the comparative contribution of turnover and nestedness to the taxonomic, functional, and phylogenetic *β*-diversity of macroinvertebrates? (ii) What is the interrelationship of these three facets of *β*-diversity? (iii) How do spatial factors, local environmental, and geo-climatic variables contribute to various facets of *β*-diversity and its components? Disturbances from human activities often lead to the homogenization of stream habitats, with species in analogous environments often sharing common traits. Organisms occupying similar ecological niches are classified as functional groups/guilds [29] and can be traced in the majority of rivers. Therefore, we hypothesized that functional *β*-diversity should be lower than taxonomic and phylogenetic *β*-diversity and be strongly influenced by nestedness (H1). The identity of species in a community depends heavily on the history of the evolution, so species replacement does not always lead to changes in functional traits. Thus, we assumed that the correlation between taxonomic and phylogenetic *β*-diversity is high, while the correlation between taxonomic and functional as well as functional and phylogenetic *β*-diversity is relatively low (H2). Given the large climatic gradient in the Hun-Tai River Basin, the strong anthropogenic influence on the local environment, and that the taxonomy of species in the community reflects the influence of the environment on traits [8], we hypothesized that geo-climatic and local environmental influence on macroinvertebrates’ *β*-diversity is greater than that of spatial factors (H3).

## 2. Materials and Methods

### 2.1. Study Area

Our study region is the Hun-Tai River Basin (40°40′–42°10′ N, 122°5′–125°17′ E) in northeastern China, with two main branches: the Hun River (415 km long) and the Tai River (413 km long), and an elevation gradient of >1000 m. The river flows through the monsoon region of a temperate zone, with an average annual precipitation of 686.4 mm and an average yearly temperature of 9 °C [18,30,31]. The Hun-Tai River Basin is an ideal area for studying *β*-diversity patterns and regimes for the following main reasons. Firstly, the gradient of anthropogenic disturbance in this area is large, with the middle and lower reaches strongly influenced by agriculture, industry, and urban construction, while the upper and source reaches are located at high altitudes in the Changbai Mountains, with high forest cover and less disturbance [31,32]. Secondly, the study area is large (27,300 km^2^) and has a large elevation gradient (over 1000 m), and such a large spatial scale helps to reveal the mechanisms of community response to environmental factors [33]. Furthermore, since the 1990s, freshwater ecosystems in the basin have gradually recovered from historic disruptions due to local government efforts (e.g., measures such as the construction of sewage systems and the closure of some coal and oil fields) [30]. However, previous investigations within this watershed have paid little attention to the diversity of river organisms’ patterns [31] and have not researched *β*-diversity and the driving factors behind it [18].

### 2.2. Field Sampling and Processing

In 2010, we collected macroinvertebrate samples from 179 sites. The sampling sites covered all wadable river sections from headwater streams to large rivers. To represent the overall community information at each sampling site, we collected macroinvertebrates from all available habitat types (e.g., cobble, rocks, fine sediments) and channel types (e.g., riffles, runs, pools) [34]. We used a GPS receiver and Trimble-Juno SB to record the geographic coordinates of each site and collected water physical information using the YSI Multiparameter instrument professional plus: dissolved oxygen (DO), pH, total dissolved solids (TDS), conductivity (Cond), and water temperature (WT). At the same time, water depth (Depth), stream width (Width), and flow velocity were measured in situ using the Global Water Flow Probe FP201. In addition, the Qualitative Habitat Evaluation Index (QHEI) was scored in situ for each site [35]. At the same time, the surface water at the sampling site was gathered and acid-fixed. Subsequently, total nitrogen (TN), ammonia nitrogen (NH3-N), soluble reactive phosphorus (PO4-P), suspended solids (SS), nitrite nitrogen (NO2-N), chemical oxygen demand (COD), and total phosphorus (TP) analyses were performed at the laboratory. TN/TP (NPR) referred to the ratio between TN and TP. For details of the sampling sites and measurements of water chemistry, refer to Qu et al. (2019) [30] and Zhou et al. (2020) [31].

A Surber net (30 × 30 cm^2^, 500 μm mesh) was used to collect macroinvertebrates. After kicking or stirring the substrate with a spatula, the samples flowed into the net in the direction of the water flow and were collected three times in duplicate. Samples were first transferred from the net to a 10 L plastic container, screened through a 40 mm mesh, and then stored in a 500 mL bottle with 70% alcohol. Identification of macroinvertebrates to the lowest taxonomic level (mainly to genus) was performed in the laboratory using the major reference books [36,37].

### 2.3. Geo-Climatic Variables

Geo-climatic variables were divided into land-use type, climate, and topography. From the Consensus Landcover Dataset, we downloaded land-use data [38]. Land-use and topographic data were based on previous studies and obtained from www.earthenv.org (accessed on 15 October 2019) [39,40]. In the original dataset, there were 12 categories of land-use cover. In our study, the category “forest” replaced evergreen broadleaf trees, deciduous broadleaf trees, evergreen/deciduous needleleaf trees, and mixed/other trees in the original data. In addition, regularly flooded shrub/herbaceous vegetation was not found in our study region. Thus, only eight categories of land-use types (i.e., open water, barren lands, snow/ice, urban, agriculture, herbaceous vegetation, shrubs, and forest) were used for the analysis (see Table S1 for details).

Moreover, 19 bioclimatic variables (Bio1 to Bio19) were obtained from the WorldClim 2 database, including isothermality, precipitation seasonality, and annual mean temperature of each sampling site [41]. Moreover, the topographic information of each site was extracted, such as aspect, slope, and elevation. The aspect indicates the north–south and east–west information for an individual site while the gradient represents the steepness of the river along the longitudinal scale [39].

### 2.4. Species Traits

All the trait information for the species was obtained exclusively from the literature [42,43,44,45]. We divided macroinvertebrates into functional feeding groups (FFG) and habit trait groups (HTG). FFGs followed the classifications proposed by Heino (2005) and included Filter-collector, Gather-collector, Shredder, Scraper, and Predator. HTGs tracked the classification of Merritt et al. (2010), which included the traits of burrowers, climbers, clingers, sprawlers, and swimmers (Table 1).

### 2.5. Data Analysis

All our data processing was done with R (Version 4.0.2, R Development Core [46]). For the description of the spatial structure of the dataset, the spatial factors were computed using the *pcnm* function in the R package *vegan* [47] according to the principal coordinates of the neighborhood matrix (PCNM) method and the distance-based Moran’s Eigenvector Maps (MEMs) using the *dbmem* function in the R package *adespatial* [48]. Since we found a significant correlation between PCNMs and MEMs (Mantel test r = 0.460, *p* = 0.001), we used MEMs in the following analyses. 

Next, we built biotic and abiotic datasets. The biotic dataset consists of (i) taxonomic *β*-diversity matrices, divided into three components (i.e., total *β*-diversity, and turnover and nestedness components with presence–absence data) with the *beta.pair* function in the R package *betapart* [49]; (ii) functional *β*-diversity was divided into the same three components (using the function *function.beta.pair* in the R package *betapart*) based on presence–absence species data and ten functional traits; (iii) phylogenetic *β*-diversity was similarly divided into three components (using the function *phylo.beta.pair* in the R package *betapart*) based on Faith’s phylogenetic diversity. 

This partitioning of *β*-diversity allowed us to understand the comparative importance of turnover and nestedness components to total taxonomic, functional, and phylogenetic *β*-diversity (question i). The abiotic datasets included (i) spatial factors (Spatial), which included 43 MEMs; (ii) geo-climatic variables (Geo), which were divided into three main categories, including 19 bioclimatic variables (Bio1-19), eight land-use types, and three topographic variables (aspect, slope, and elevation); and (iii) local environmental variables (Local), including 18 physicochemical variables for both field and laboratory measurements (Table S1).

The function *mantel* in the R package *vegan* was used to perform the Mantel test for taxonomic, functional, and phylogenetic *β*-diversity to determine the correlation between them (question ii). Tests of significance were performed with 999 permutations. The relevance of two heterogeneity or distance matrices is represented by the statistic r (range, −1 to 1) in the Mantel test. We next carried out a distance-based redundancy analysis (db-RDA) of each facet of *β*-diversity and its components [50]. This was in order to determine whether *β*-diversity was influenced by abiotic factors (i.e., spatial factors, geo-climatic, and local environmental). Before analysis, we deleted the variables with significant multicollinearity (with variance inflation factor ≥ 3; *vifstep* function in the R package *usdm*) [51] in all three datasets (i.e., Spatial, Geo, and Local). We used the function *capscale* in the R package *vegan* to include variables that did not display significant collinearity in db-RDA. We tested the general significance of the ranking schemes, the amount of explained variation (R^2^), and the marginal significance of each variable included in the model. Similar to prior studies, we corrected for negative eigenvalues in all db-RDA analyses using *sqrt.dist* in R [18,52]. 

We used the variance partitioning analysis (VPA) approach to the quantification of the comparative contributions of the three abiotic factors to every facet and component of *β*-diversity (question iii), which has been commonly applied to determine ecological processes [53,54]. To obtain the final dataset of Spatial, Geo, and Local, we used *forward.sel* function in the R package *adespatial* for forward selection, and two stopping criteria were set: the adjusted coefficient of determination (adjusted R^2^) and significance level [55]. At a significance level of α = 0.05, the significance of the pure fractions was examined with the *anova* function in the R package *vegan*. VPA was performed using the *varpart* function in the R package *vegan*. The work flowcharts for the three facets of *β*-diversity are shown in Figure S1–S3.

## 3. Results

### 3.1. Taxonomic, Functional, and Phylogenetic β-Diversity Components

**Figure 1:**
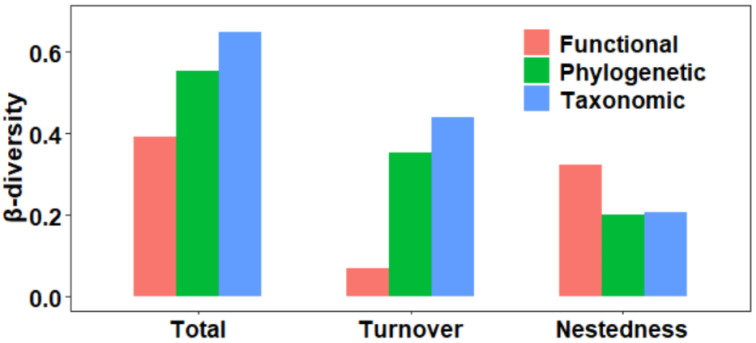
The three facets (taxonomic, functional, and phylogenetic) of macroinvertebrates’ *β*-diversity and its components (i.e., total, turnover, and nestedness) in the Hun-Tai River Basin.

A total of 162 macroinvertebrate species were observed in this study (see Table S2 for a species list), with a mean species richness of 14.56 (range 1–48) per site. Taxonomic total *β*-diversity was highest (0.649 ± 0.147) and mostly contributed by turnover (0.441 ± 0.183) and to a lesser extent by nestedness (0.208 ± 0.164). For phylogenetic *β*-diversity, the mean values for the three components were relatively low (0.552 ± 0.138 for total, 0.352 ± 0.171 for turnover, and 0.200 ± 0.157 for nestedness, respectively). For functional *β*-diversity, the lowest mean values were found for the three components (0.393 ± 0.304 for total, 0.071 ± 0.176 for turnover, and 0.322 ± 0.285 for nestedness), and the nestedness contributed significantly more to the total functional *β*-diversity than the turnover (Figure 1). This exactly proved H1.

### 3.2. Correlation of Taxonomic, Functional, and Phylogenetic β-Diversity

**Figure 2:**
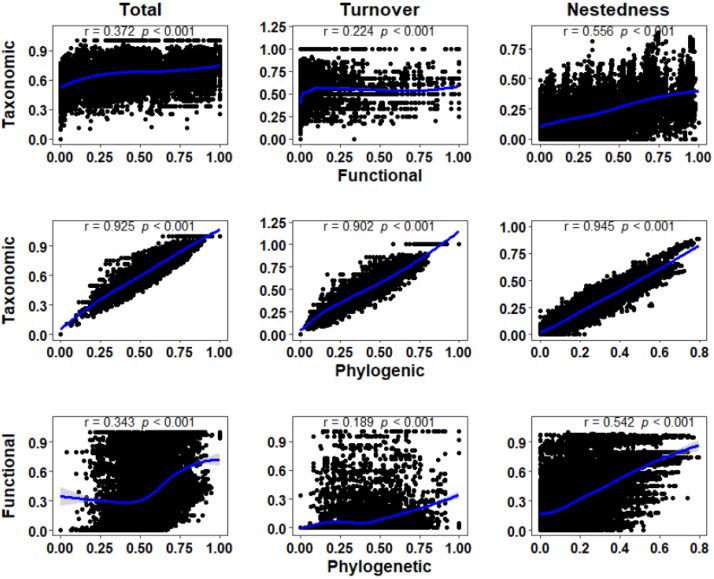
The relationships between taxonomic, functional, and phylogenetic *β*-diversity components for macroinvertebrates (i.e., total *β*-diversity, turnover, and nestedness). According to the Mantel test, these relationships were statistically significant (*p* < 0.001). The Mantel coefficient is represented by the r correlation shown in the figure. The 95% confidence interval of the fit is indicated by the gray shaded area and the LOESS smoothing is indicated by the blue solid line.

The strongest relationship between taxonomic and functional *β*-diversity components was found with the Mantel test (*p* < 0.001), with the highest Mantel correlation coefficient of nestedness (r = 0.945), followed by total *β*-diversity (r = 0.925), and turnover (r = 0.902). The correlation between taxonomic and functional *β*-diversity components was relatively weak (*p* < 0.001), with the highest Mantel correlation coefficient of nestedness (r = 0.556), followed by total *β*-diversity (r = 0.372), and turnover (r = 0.224). The weakest correlation was found between functional and phylogenetic *β*-diversity, with the highest Mantel correlation coefficient of nestedness (r = 0.542), followed by total *β*-diversity (r = 0.343), and turnover (r = 0.189) (Figure 2). This supported H2.

### 3.3. Main Drivers of Taxonomic, Functional, and Phylogenetic β-Diversity Components

Generally, the selection of variables in the db-RDA model was different for various components and facets of *β*-diversity. For the different components of taxonomic *β*-diversity, 7, 4 and 3 Local; 6, 2, and 4 Geo along with 16, 9 and 15 Spatial were finally selected for total *β*-diversity, turnover, and nestedness, respectively, by a forward selection procedure and multicollinearity test (Table 2). For the functional *β*-diversity component, 6, 3 and 6 Local; 4, 2 and 4 Geo along with 15, 9 and 14 Spatial were finally selected for total *β*-diversity, turnover, and nestedness, respectively (Table 3). In addition, for the phylogenetic *β*-diversity component, 5, 3 and 4 Local; 6, 3 and 5 Geo along with 18, 6 and 14 Spatial were selected in terms of total *β*-diversity, turnover, and nestedness respectively (Table 4).

**Figure 3:**
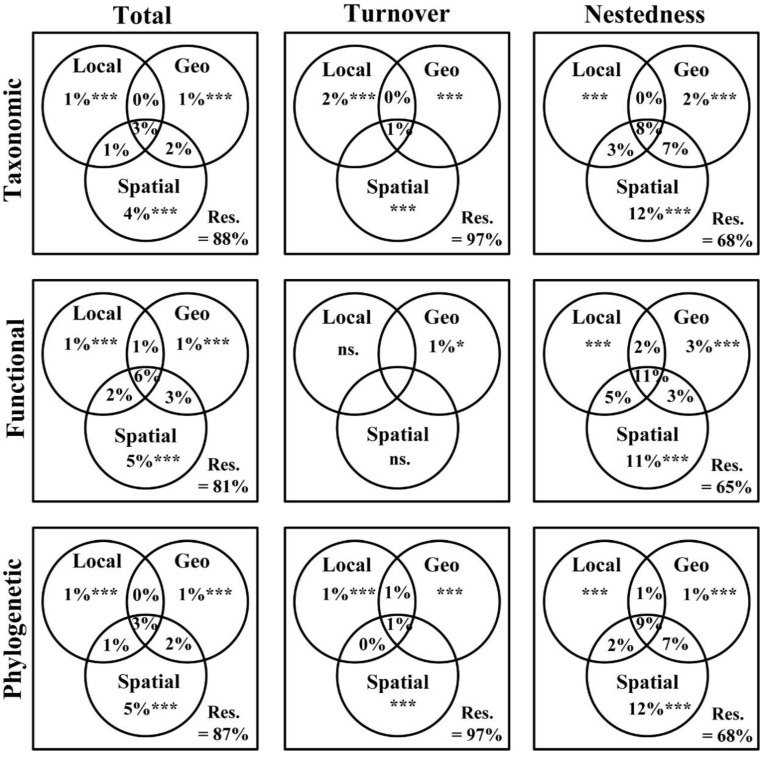
Relative importance of local environmental (Local), geo-climatic (Geo), and spatial (Spatial) factors on taxonomic, functional, and phylogenetic *β*-diversity components (i.e., total, turnover, and nestedness) of macroinvertebrates. Each set of plots indicates the pure effects of the Local, Geo, and Spatial factors on different facets and components of *β*-diversity (i.e., considering the effect of only one factor alone), the interaction between two variables (Local*Geo, Local*Spatial, Geo*Spatial), the joint effect of the three factors, and the unexplained effect (i.e., Res.) (total variation = 100). Values indicate the adjusted R^2^ (values < 0 are not shown). No significant (ns.) indicates no value for the functional turnover. Significance was indicated as *** *p* < 0.001, * *p* < 0.05.

The VPA showed that among the three component facets of *β*-diversity, Local, Geo, and Spatial had slightly different pure and shared components (Figure 3). Overall, Local (1% to 2%) has the lowest pure contribution, lower than Geo (1–3%) and Spatial (4–12%). Moreover, among the taxonomic *β*-diversity components, the shared effects of Local, Geo, and Spatial on total, turnover, and nestedness were 3%, 1%, and 8%, respectively. For functional *β*-diversity, the shared effects on total and nestedness were 6% and 11%. And for phylogenetic *β*-diversity, the shared effects on total, turnover, and nestedness were 3%, 1%, and 9%. This suggested a significant interaction among the three abiotic factors. For taxonomic *β*-diversity, the significant global models explained 12% of the total, 3% of turnover, and 32% of nestedness. For functional *β*-diversity, the significant global models explained 19% of total *β*-diversity and 35% of nestedness (no Local and Spatial models were found to be significant for turnover). For phylogenetic *β*-diversity, the significant global models explained 13% of the *β*-diversity, 3% of the turnover, and 32% of the nestedness.

## 4. Discussion

### 4.1. Contribution of Turnover and Nestedness

For exploring the relative importance of various components of macroinvertebrates’ *β*-diversity to taxonomic, functional, and phylogenetic *β*-diversity, we partitioned *β*-diversity into three components (i.e., total *β*-diversity, turnover, and nestedness). We observed that taxonomic and phylogenetic total *β*-diversity was higher than functional total *β*-diversity and mainly derived from the contribution of turnover. This finding was similar to the results of previous studies on macroinvertebrates, all of which have reported high taxonomic *β*-diversity [8,28,52]. This suggested that the taxonomic and phylogenetic compositions of macroinvertebrate assemblages vary considerably between sites. There are several possible reasons for the high taxonomic and phylogenetic turnover in our study area. First, the Hun-Tai River Basin is large (27,300 km^2^) and the upstream areas flow through mountainous areas, with a high diversity of mountain species and a clear spatial and climatic gradient between sites, resulting in high taxonomic and phylogenetic turnover [8,28]. Second, anthropogenic disturbances from industry, agriculture, and extraction have drastically altered the whole context and stream habitats of the Hun-Tai River Basin [30,56]. Human activity has previously been documented as a key cause of strong environmental gradients in the study of river systems, which also provides a strong force for the classification of riverine species [57,58,59]. This may have profound implications for the distribution patterns and phylogeny of current macroinvertebrate communities. Changes in the assemblage of local macroinvertebrate species may be attributed to different evolutionary and adaptive tactics in response to different environments, leading to a significantly high turnover between sites.

By contrast, total functional *β*-diversity and its turnover component were much lower, which validated H1. This suggested that most of the functional traits of the macroinvertebrates in our study area are shared among different sites, which is consistent with previous studies [18,60]. Low values of functional total *β*-diversity may be the consequence of functional convergence, with the adaptation of different species to comparable habitat situations [61]. Thus, different species have the same traits at different stream sites, resulting in low functional variability between stream sites [8]. One of the potential reasons for the significant contribution of nestedness to functional *β*-diversity is selective extinction [17]. For example, environmental filtering led to some functional traits being more common than others. In addition, habitat heterogeneity across sites in the study area may also lead to high nestedness and low turnover. For instance, certain locations with high habitat heterogeneity may have species with a variety of functional traits, while certain species with specific functions are only present in certain homogenized habitats [62]. Communities with multiple functional traits contain communities with fewer traits [8]. Considering the strong historic disturbances that have occurred in our study basin, homogenization of macroinvertebrates’ habitats and biological traits may have occurred and still have an impact on the current functional trait composition. In order to more comprehensively conserve biodiversity and manage streams, maintaining habitat heterogeneity is essential to improving functional biodiversity in our study area [18].

### 4.2. Relationships between Taxonomic, Functional, and Phylogenetic β-Diversity

We found weak correlations of functional with both taxonomic and phylogenetic *β*-diversity (Mantel correlation coefficients ranged between 0.024–0.556 and 0.189–0.542 respectively). Not surprisingly, this agrees with previous studies of stream macroalgae and macroinvertebrates in freshwater ecosystems [28,63]. Hence, we can infer that taxonomic and functional *β*-diversity components along with functional and phylogenetic *β*-diversity might provide complementary information. Such results indicated that species with different taxonomic and phylogenetic information in the study area shared the same traits, which further emphasizes the importance of studying functional traits.

Interestingly, we found strong correlations between taxonomic and phylogenetic *β*-diversity components, and their Mantel correlation coefficients ranged between 0.902–0.945 (Figure 2). Phylogeny is indispensable in community ecology because it links ecological models to the evolutionary mechanisms behind diversity and trait variation [64,65]. Hypothetically influenced by intense phylogenetic signals, species that were closely associated tended to be more similar than species that were remotely related. Therefore, they would be more likely to occur within a community [65]. The main contributor to taxonomic and phylogenetic *β*-diversity in our study was turnover (0.441 ± 0.183 and 0.352 ± 0.171, respectively). Hence, we can infer that the taxonomic diversity of the study area may demonstrate significant phylogenetic diversity, which made it easy to interpret the strong correlation between the two [26]. However, few studies have concluded that there is a high correlation between macroinvertebrates’ taxonomic and phylogenetic *β*-diversity, and the reasons for their high correlation are subject to further study.

### 4.3. Main Drivers of Taxonomic, Functional, and Phylogenetic β-Diversity

It is well known that the biodiversity pattern of stream ecosystems is impacted by a variety of factors ranging across local to regional scales [66,67,68]. Consideration of the spatial scale of the study region [69] and different facets of biodiversity [66] may influence the relative importance of these factors. The objective of our research was to uncover the relative effects of spatial factors, local environment, and geo-climate on macroinvertebrate taxonomic, functional, and phylogenetic *β*-diversity. The VPA outcomes (Figure 3) showed that the total and nestedness components of taxonomic, functional, and phylogenetic *β*-diversity were largely affected by spatial factors but weakly influenced by local environment and geo-climate, which does not support H3. From a metacommunity theory perspective, changes in community composition may be influenced by dispersal limitations or mass effects [70]. Mass effect refers to the fact that species can survive under unfavorable conditions through immigration [71], while dispersal limitation is defined as the inability of species to achieve suitable habitats for survival owing to poor dispersal rates or physical obstacles [72]. At relatively broad spatial scales, species have also been found to be potentially influenced by dispersal dynamics [2], as described in this study. The most obvious point in our findings is the significance of spatial factors in explaining the total and nestedness components of taxonomic, functional, and phylogenetic *β*-diversity. Based on theoretical experience, spatial factors associated with dispersal limitation may have a significant effect in driving macroinvertebrate community composition over large spatial extents [67,73]. Therefore, due to the large spatial scale of the region of our study, dispersal limitation has probably contributed to some extent to the variation in macroinvertebrates’ *β*-diversity.

Furthermore, despite the high residuals of 65–97% for our VPA (Figure 3), the explanatory ability is comparable to relevant studies [28,52,74]. From other studies, we realized that there are many more variables affecting spatial *β*-diversity not considered in our study, such as, for example, flow regimes [75], metal contamination [76], and polycyclic aromatic hydrocarbons [56]. Of particular note is the fact that the study basin is severely impacted by the extraction and metals industry, and this significantly impacts benthic organisms such as macroinvertebrates and algae [76]. Therefore, the spatial patterns derived from our findings may be influenced by metal contamination. Furthermore, if further study includes an analysis of the above factors, it may enable a more effective interpretation of findings, thus giving us a clearer view of the influence of the environment on *β*-diversity.

We also found that geo-climate accounted for a significant fraction of the statistics for taxonomic, functional, and phylogenetic *β*-diversity components (in addition to the turnover component of taxonomic and phylogenetic *β*-diversity). The importance of environmental effects on macroinvertebrate *β*-diversity has also been highlighted in previous studies [28,52,77]. In general, changes in climatic factors can cause changes in environmental situations that can affect macroinvertebrate communities [52,68]. For instance, temperature increases and decreases can affect the growth of aquatic organisms and indirectly limit the distribution of species in the landscape by affecting water temperature [78]. Similarly, changes in stream flow are largely influenced by variations in precipitation, which further affects biological community composition [52].

### 4.4. Management Implications and Conclusions

Following the metacommunity theory, spatial processes and environmental effects together influence the composition of communities [2,72]. Environmental filtering comes into play only after species have spread to new habitats [79]. Unexpectedly, few previous studies have revealed the impact of multi-scale environments on macroinvertebrates’ *β*-diversity patterns based on these three facets of *β*-diversity (taxonomic, functional, and phylogenetic) at the same time [52]. Previous *β*-diversity-based studies have involved many different species, including birds, soil animals, and mammals [80,81,82]. However, traditionally, changes in community composition have been examined on the basis of species identity [2,22], and this is a neglect of functional and phylogenetic variation among species. Therefore, community ecologists have recently argued that communities should be understood not only at the species level but also by considering functional and phylogenetic methods, to better understand how different factors influence *β*-diversity [6,7,8,27]. Integrating the connections between species, functional traits and phylogeny can provide valuable additional perspectives for understanding the drivers of community composition [6,27]. Hence, studying patterns of functional *β*-diversity can provide complementary information for taxonomic *β*-diversity and can help decision-makers to develop area-specific biodiversity conservation strategies and carry out recovery estimates [54,61]. For example, when nestedness contributes significantly to taxonomic total *β*-diversity, streams with high diversity should be prioritized for protection, and when turnover makes a significant contribution to total taxonomic *β*-diversity, the entire basin should be protected at the same time [23].

Although high total and turnover rates of taxonomic and phylogenetic *β*-diversity were obtained in our study, low turnover and high nestedness rates of functional *β*-diversity suggest that functional biodiversity may not have recovered at many sites. Our results suggested that the convergence of functional traits among macroinvertebrate communities might be a consequence of past anthropogenic impacts (e.g., habitat homogenization due to disturbance). Therefore, environmental assessments in this watershed should consider taxonomic, functional, and phylogenetic approaches. In addition, temporal scales can be introduced to allow for more accurate comprehension of the mechanisms that shape *β*-diversity over time, to guide biodiversity conservation in specific areas [11,83,84].

In summary, the partitioning of *β*-diversity into different components (turnover and nestedness) and the study of different facets (taxonomic, functional, and phylogenetic) of *β*-diversity are emerging as popular approaches in biodiversity studies, as such studies offer different perspectives on community assembly mechanisms [18,77,85]. Within our study, we analyzed the relevance and main drivers among these facets and components of *β*-diversity (i.e., spatial factors, local environment, and geo-climate) based on these perspectives that would not be revealed if only taxonomic level information had been considered [86]. This is because the distribution of species and ecological communities is motivated by a variety of evolutionary and ecological mechanisms that function at numerous spatial and temporal scales [21]. Considering that community assembly mechanisms are highly complex, we recommend an approach that integrates the three facets (i.e., taxonomic, functional, and phylogenetic) of diversity in conducting biodiversity assessment and restoration efforts. Therefore, this approach allows for a more comprehensive insight into the mechanisms by which multiple factors influence different facets of biodiversity, rather than focusing solely on the taxonomic facet.

## Figures and Tables

**Table 1:** Macroinvertebrates’ traits, their descriptions, codes, and categories used in this study.

	Categories	Codes	Descriptions
1. Functional feeding groups [43]	Filter-collector	FFG_Fil	The FFG classification is based on the food consumed and also considers the morphological and behavioral characteristics used in the food acquisition [45]
	Gather-collector	FFG_Gat	
	Shredder	FFG_Shr	
	Scraper	FFG_Scr	
	Predator	FFG_Pre	
2. Habit trait groups [44]	Burrower	HTG_Bur	The HTG describe the mobility and microhabitat use of the macroinvertebrates [44]
	Climber	HTG_Clim	
	Clinger	HTG_Clin	
	Sprawler	HTG_Spr	
	Swimmer	HTG_Swi	

**Table 2:** The local environmental (Local), geo-climatic (Geo), and spatial (Spatial) variables that were selected for the taxonomic *β*-diversity component, respectively. Each variable is displayed in the order in which it was selected. F, *p,* and AdjR^2^Cum values are displayed. None of the selected variables showed significant multicollinearity (coefficient of variance inflation < 3). Significance is indicated as *** *p* < 0.001.

Total				Turnover				Nestedness			
Variables	AdjR^2^Cum	F	*p*	Variables	AdjR^2^Cum	F	*p*	Variables	AdjR^2^Cum	F	*p*
Local ***				Local ***				Local ***			
Depth	0.073	13.981	0.001	Depth	0.018	4.059	0.009	Depth	0.080	15.372	0.001
WT	0.089	3.913	0.005	WT	0.032	3.387	0.012	PO4	0.103	5.213	0.006
Width	0.103	3.626	0.008	Width	0.049	3.919	0.004	NPR	0.114	3.126	0.036
PO4	0.114	3.090	0.014	NPR	0.058	2.497	0.031				
NPR	0.131	4.157	0.002								
pH	0.142	3.016	0.020								
Velocity	0.152	2.886	0.024								
Geo ***				Geo ***				Geo ***			
Elevation	0.094	18.125	0.001	Elevation	0.023	4.827	0.001	Elevation	0.118	23.133	0.001
Bio15	0.127	7.319	0.001	Bio15	0.045	4.800	0.001	Bio15	0.166	10.516	0.001
Shrubs	0.139	3.335	0.010					Shrubs	0.183	4.435	0.011
Herbaceous	0.150	3.016	0.022					Snow.ice	0.192	2.826	0.044
Urban	0.167	4.365	0.009								
Bio3	0.175	2.489	0.027								
Spatial ***				Spatial ***				Spatial ***			
MEM3	0.037	7.449	0.001	MEM40	0.023	4.974	0.001	MEM3	0.076	14.710	0.001
MEM2	0.067	6.149	0.001	MEM6	0.040	3.810	0.002	MEM6	0.122	9.581	0.001
MEM4	0.091	5.371	0.001	MEM3	0.054	3.503	0.007	MEM8	0.157	7.854	0.003
MEM7	0.114	5.210	0.002	MEM4	0.064	2.731	0.023	MEM11	0.181	5.737	0.004
MEM9	0.136	5.201	0.001	MEM9	0.074	2.693	0.019	MEM5	0.202	5.333	0.003
MEM15	0.158	5.130	0.001	MEM24	0.082	2.400	0.029	MEM1	0.223	5.200	0.005
MEM1	0.178	4.876	0.002	MEM8	0.089	2.205	0.044	MEM15	0.240	4.757	0.005
MEM5	0.194	4.225	0.004	MEM35	0.095	2.144	0.046	MEM7	0.255	4.164	0.013
MEM6	0.209	4.035	0.008	MEM1	0.102	2.151	0.036	MEM2	0.270	4.247	0.010
MEM16	0.223	3.883	0.004					MEM9	0.283	3.688	0.022
MEM8	0.235	3.414	0.010					MEM16	0.294	3.435	0.028
MEM18	0.246	3.249	0.010					MEM18	0.304	3.263	0.033
MEM11	0.256	3.015	0.005					MEM4	0.313	3.189	0.032
MEM28	0.263	2.452	0.029					MEM29	0.323	3.121	0.023
MEM31	0.269	2.340	0.041					MEM35	0.330	2.728	0.037
MEM40	0.275	2.247	0.035								

Bio3 = Isothermality, Bio15 = Precipitation Seasonality.

**Table 3:** The local environmental (Local), geo-climatic (Geo), and spatial (Spatial) variables that were selected for the functional *β*-diversity component, respectively. Each variable is displayed in the order in which it was selected. F, *p,* and AdjR^2^Cum values are displayed. None of the selected variables showed significant multicollinearity (coefficient of variance inflation < 3). Significance is indicated as * *p* < 0.05, *** *p* < 0.001.

Total				Turnover				Nestedness			
Variables	AdjR^2^Cum	F	*p*	Variables	AdjR^2^Cum	F	*p*	Variables	AdjR^2^Cum	F	*p*
Local ***				Local				Local ***			
Depth	0.095	18.413	0.001	Depth	0.041	8.048	0.001	Depth	0.097	18.860	0.001
PO4	0.162	14.293	0.001	PO4	0.065	5.348	0.006	PO4	0.161	13.550	0.001
SS	0.190	6.684	0.011	Width	0.091	5.617	0.002	SS	0.190	6.903	0.004
Velocity	0.208	4.608	0.017					Velocity	0.208	4.684	0.017
NPR	0.224	4.426	0.031					NPR	0.224	4.401	0.027
pH	0.246	5.575	0.006					pH	0.246	5.588	0.007
Geo ***				Geo *				Geo ***			
Elevation	0.132	26.217	0.001	Elevation	0.058	11.223	0.001	Elevation	0.132	26.185	0.001
Bio15	0.185	11.764	0.001	Bio15	0.110	10.589	0.001	Bio15	0.188	12.388	0.001
Urban	0.210	6.132	0.011					Urban	0.212	6.145	0.009
Barrenlands	0.222	3.523	0.030					Barrenlands	0.225	3.708	0.034
Spatial ***				Spatial				Spatial ***			
MEM6	0.057	10.946	0.001	MEM3	0.037	7.457	0.001	MEM3	0.061	11.706	0.001
MEM3	0.112	11.363	0.001	MEM9	0.060	5.035	0.003	MEM6	0.117	11.520	0.001
MEM5	0.151	8.413	0.001	MEM1	0.081	4.674	0.009	MEM9	0.154	8.279	0.002
MEM9	0.186	8.063	0.005	MEM5	0.100	4.520	0.005	MEM5	0.192	8.569	0.002
MEM1	0.217	7.350	0.004	MEM24	0.112	3.158	0.029	MEM1	0.226	8.059	0.001
MEM10	0.235	4.853	0.015	MEM6	0.124	3.176	0.022	MEM10	0.244	4.938	0.018
MEM15	0.251	4.359	0.031	MEM8	0.135	2.934	0.035	MEM29	0.262	4.934	0.014
MEM7	0.265	4.087	0.028	MEM29	0.144	2.675	0.035	MEM7	0.277	4.318	0.028
MEM29	0.278	3.949	0.032	MEM31	0.151	2.461	0.039	MEM15	0.292	4.301	0.029
MEM31	0.292	3.946	0.031					MEM8	0.307	4.309	0.024
MEM24	0.305	3.992	0.027					MEM31	0.319	3.822	0.031
MEM8	0.318	4.054	0.032					MEM4	0.331	3.832	0.034
MEM4	0.332	4.044	0.033					MEM16	0.343	3.820	0.027
MEM16	0.344	3.821	0.033					MEM11	0.355	3.705	0.032
MEM11	0.356	3.800	0.033								

Bio15 = Precipitation Seasonality.

**Table 4:** The local environmental (Local), geo-climatic (Geo), and spatial (Spatial) variables that were selected for the phylogenetic *β*-diversity component, respectively. Each variable is displayed in the order in which it was selected. F, *p,* and AdjR^2^Cum values are displayed. None of the selected variables showed significant multicollinearity (coefficient of variance inflation < 3). Significance is indicated as *** *p* < 0.001.

Total				Turnover				Nestedness			
Variables	AdjR^2^Cum	F	*p*	Variables	AdjR^2^Cum	F	*p*	Variables	AdjR^2^Cum	F	*p*
Local ***				Local ***				Local ***			
Depth	0.079	15.278	0.001	Depth	0.024	5.150	0.003	Depth	0.077	14.813	0.001
PO4	0.094	3.731	0.012	Width	0.039	3.595	0.007	PO4	0.107	6.654	0.002
NPR	0.113	4.476	0.005	WT	0.050	2.827	0.015	NPR	0.128	4.819	0.014
Width	0.129	3.985	0.004					pH	0.138	2.998	0.048
pH	0.137	2.533	0.050								
Geo ***				Geo ***				Geo ***			
Elevation	0.110	21.563	0.001	Elevation	0.031	6.252	0.001	Elevation	0.122	24.071	0.001
Bio15	0.140	6.766	0.001	Bio15	0.050	4.400	0.004	Bio15	0.174	11.407	0.001
Herbaceous	0.153	3.445	0.007	Herbaceous	0.060	2.654	0.029	Shrubs	0.189	3.976	0.014
Urban	0.166	3.519	0.011					Urban	0.200	3.249	0.038
Shrubs	0.181	4.093	0.007					Bio3	0.209	2.839	0.050
Bio3	0.190	2.661	0.033								
Spatial ***				Spatial ***				Spatial ***			
MEM3	0.042	8.284	0.001	MEM40	0.030	6.201	0.001	MEM3	0.086	16.541	0.001
MEM4	0.066	5.276	0.004	MEM3	0.047	3.816	0.004	MEM6	0.133	10.035	0.001
MEM2	0.090	5.323	0.003	MEM6	0.063	3.819	0.003	MEM11	0.163	6.948	0.004
MEM7	0.114	5.448	0.002	MEM4	0.073	2.795	0.014	MEM8	0.194	7.115	0.003
MEM15	0.137	5.162	0.002	MEM35	0.082	2.514	0.028	MEM5	0.223	7.137	0.003
MEM6	0.156	4.628	0.001	MEM7	0.089	2.358	0.023	MEM1	0.243	5.305	0.007
MEM1	0.173	4.403	0.002					MEM15	0.261	4.868	0.009
MEM9	0.189	4.151	0.002					MEM2	0.279	4.948	0.011
MEM16	0.204	3.933	0.009					MEM9	0.293	4.210	0.019
MEM5	0.219	4.004	0.004					MEM7	0.307	4.124	0.013
MEM11	0.232	3.657	0.007					MEM18	0.321	4.094	0.024
MEM18	0.244	3.533	0.010					MEM16	0.334	4.010	0.020
MEM8	0.255	3.090	0.015					MEM10	0.344	3.428	0.033
MEM40	0.263	2.712	0.016					MEM40	0.352	2.941	0.046
MEM29	0.271	2.681	0.023								
MEM28	0.277	2.342	0.030								
MEM31	0.284	2.358	0.043								
MEM37	0.290	2.336	0.049								

Bio3 = Isothermality, Bio15 = Precipitation Seasonal.

## Data Availability

The data will be available on request.

## Supplementary Materials

The following supporting information can be downloaded at: https://www.mdpi.com/article/10.3390/ani12192648/s1, Table S1: Summary of local environmental (Local), geo-climatic (Geo) and spatial (Spatial) variables with their codes and descriptions in this study; Table S2: Species list that observed in this study; Figure S1: A flow-chart of taxonomic *β*-diversity analyses; Figure S2: A flow-chart of functional *β*-diversity analyses; Figure S3: A flow-chart of phylogenetic *β*-diversity analyses.
