# Supplementary material for: Spatial Factors Outperform Local Environmental and Geo-Climatic Variables in Structuring Multiple Facets of Stream Macroinvertebrates’ β-Diversity
Source: Animals (Basel). 2022 Oct 2;12(19):2648. doi: 10.3390/ani12192648 (PMC9558512; doi:10.3390/ani12192648)
Supplement: Supplementary file 1 [file animals-12-02648-s001.zip › animals-1927422-supplementary.pdf]

Table S1. Summary of local environmental (Local), geo-climatic (Geo) and spatial (Spatial) variables with their codes and descriptions in this study.

Variables					
Code	Unit	Description	Mean	Min	Max
**Local**		**Local environmental variables**			
pH	-	pH	8.55	6.73	10.12
DO	mg/L	Dissolved oxygen	9.18	0.01	15.63
Cond	μs/cm	Conductivity	249.62	3.90	1124.00
SS	mg/L	Suspended solid	37.36	1.00	1110.00
TDS	mg/L	Total dissolved solid	201.65	14.95	903.00
COD	mg/L	Chemical oxygen demand	14.78	0.00	84.65
WT	◦C	Water temperature	16.29	7.20	26.00
CODMn	mg/L	Permanganate index of COD	5.02	0.00	45.30
NH3-N	mg/L	Ammonia nitrogen	1.05	0.00	14.50
NO2-N	mg/L	Nitrite nitrogen	0.15	0.00	2.23
PO4-P	mg/L	Soluble reactive phosphorus	0.07	0.00	0.93
TN	mg/L	Total Nitrogen	6.30	0.48	22.60
TP	mg/L	Total Phosphorus	0.17	0.00	2.55
NPR	-	TN/TP	382.43	0.00	3986.00
Width	m	Width	48.78	1.50	420.00
Velocity	m/s	Velocity	0.47	0.00	1.14
Depth	cm	Water depth	25.96	6.33	130.00
QHEI	-	Habitat score	112.38	51.00	185.00
**Geo**		**Geo-climatic variables**			
Forest	%	Forest%	67.35	5.00	100.00
Shrubs	%	Shrubs%	1.81	0.00	8.00
Herbaceous	%	Herbaceous vegetation%	0.34	0.00	6.00
Agriculture	%	Cultivated and managed vegetation%	24.17	0.00	88.00
Urban	%	Urban/built-up%	0.97	0.00	17.00
Snow.ice	%	Snow/ice%	0.36	0.00	20.00
Barrenlands	%	Barren lands/sparse vegetation%	4.77	0.00	19.00
Water	%	Open water%	0.23	0.00	1.00

Elevation	m	Elevation of the sampling site	198.26	3.83	555.00
Slope	◦	Slope of the sampling site	3.35	0.13	24.39
Aspect	◦	Aspect of the sampling site	180.57	2.07	354.81
Bio1	◦C	Annual Mean Temperature	7.44	5.19	9.00
Bio2	◦C	Mean Diurnal Range	11.51	9.72	12.75
Bio3	-	Isothermality	24.65	22.28	25.80
Bio4	-	Temperature Seasonality	1270.15	1200.55	1375.42
Bio5	◦C	Max Temperature of Warmest Month	29.26	27.50	30.50
Bio6	◦C	Min Temperature of Coldest Month	-17.44	-22.00	-13.30
Bio7	◦C	Temperature Annual Range	46.70	43.10	51.00
Bio8	◦C	Mean Temperature of Wettest Quarter	22.25	20.07	23.78
Bio9	◦C	Mean Temperature of Driest Quarter	-9.27	-12.92	-7.03
Bio10	◦C	Mean Temperature of Warmest Quarter	22.25	20.07	23.78
Bio11	◦C	Mean Temperature of Coldest Quarter	-9.27	-12.92	-7.03
Bio12	mm	Annual Precipitation	772.15	623.00	942.00
Bio13	mm	Precipitation of Wettest Month	203.54	167.00	250.00
Bio14	mm	Precipitation of Driest Month	6.70	4.00	10.00
Bio15	-	Precipitation Seasonality	103.51	98.37	108.11
Bio16	mm	Precipitation of Wettest Quarter	488.01	397.00	595.00
Bio17	mm	Precipitation of Driest Quarter	24.85	16.00	35.00
Bio18	mm	Precipitation of Warmest Quarter	488.01	397.00	595.00
Bio19	mm	Precipitation of Coldest Quarter	24.85	16.00	35.00
**Spatial**		**Spatial factors**			
MEM1	-	43 distance-based Moran's eigenvector maps (MEM1-MEM43)	0	-1.208	2.133
MEM2	-		0	-2.306	2.012
…	-		…	…	…
MEM43	-		0	-3.407	3.395

Variables indicating significant multicollinearity (with variance inflation factor >=3) are excluded. For spatial variables, only MEMs with positive eigenvalues are retained as spatial explanatory variables.

Table S2. Species list that observed in this study.

Species name
Acanthomysis sp.
Acentrella sibirica
Agriotypus sp.
Allocosmoecus sp.
Amphinemura sp.
Ampumixis sp.
Anisogomphus(maacki)
Anodonta woodiana
Antocha sp.
Arctopsyche sp.
Argyroneta aquatica
Atherix sp.
Baetiella japonica
Baetis bicaudatus
Beatis sp.1
Beatis sp.2
Beatis thermicus
Bellamya aeruginosa
Berosus sp.
Bezzia sp.
Bithynia fuchsiana
Branchiura sowerbyi Beddard
Caenis sp.
Carabidae
Ceratopogoniidae sp.1
Ceratopogoniidae sp.2
Cercion sexlineatum
Cheumatopsyche sp.1
Cheumatopsyche sp.2
Chironominae
Choroterpes altioculus
Cincticostella orientalis
Cinygmula sp.
Clinocera sp.
Crambidae
Crocothemis sp.
Davidius moiwanus
Diamesinae
Dicronota sp.
Discocerina sp.
Dolichocephala
Dolichopodidae

Dolichopus sp.
Donacia sp.
Drunella basalis
Drunella trispina
Dugesia sp.
Dytiscidae
Ecdyonurus bajkovae
Ecdyonurus kibunensis
Ecdyonurus sp.1
Ecdyonurus sp.2
Ecdyonurus tobiironis
Ecdyonurus viridis
Ecnonumus sp.
Elmidae
Elophila sp.1
Elophila sp.2
Epeorus latifolium
Ephemera orientalis
Ephemera strigata
Ephemerella atagosana
Ephydra sp.
Ephydridae
Eubasilissa sp
Eubrianax
Gammarus sp.
Glossosoma altaicum
Glossosoma sp.
Goera japonica
Gyraulus compressus
Gyraulus convexiusculus
Gyretes sp.
Hagenella sp.
Haliplus sp.
Hemerodromia sp.
Hexatoma sp.
Homphylax sp.
Hybomitra montana
Hydatophylax festivus
Hydrobius sp.
Hydrocyphon sp.
Hydrophilidae
Hydrophorus sp.
Hydropsyche kozhantschikovi
Hydropsyche nevae

Hydropsyche orientalis
Hydropsyche sp.
Hydropsyche yaeyamensis
Hydroptila sp.
Isonychia japonica
Isoperla sp.
Lepidostoma sp.
Lethocerus indicus
Leuctuidae
Limnephilidae
Limnephilus sp.
Liodessus sp.
Matrona cornelia
Megrcys ochracea
Molannodes
Nematoda
Neonectes natrix
Neoperla sp.
Nephelopsis sp.
Nihomogomphus(viridis)
Odontomyia sp.
Oligochaeta
Onychogomphus(viridicostus)
Oreogeton sp.
Oreoleptidae
Orientalis
Ormosia sp.
Orthocladinae
Oyamia sp.1
Oyamia sp2.
Paraleptophlebia japonica
Paraleptophlebia westoni
Parapoynx crisonalis
Perlomyia
Phanocelia
Placobdella sp.
Platycnemis sp.
Potamanthus huoshanensis
Prosimulium daisetsense
Prosimulium sp.
Protohermes grandis
Psilotreta kisoensis
Psychoda sp.
Psychomyia sp.

Radix auracularis
Radix swinhoei
Rhithorogena sp.
Rhyacophila brevicephala
Rhyacophila kawamurae
Rhyacophila nigrocephala
Rhyacophila sibirica
Rhyacophila sp.1
Rhyacophila sp.2
Rhyphium sp.
Scatella sp.
Serratella rufa
Serratella setigera
Setodes turbatus (Ceraclea)
Sieboldius sp.
Simulium japonicum
Simulium sp.
Simulium yonagoense
Siphlonurus sp.
Sperchopsis sp.
Stavsolus sp.
Stenopsyche marmorata
Stylurus sp.
Suwallia sp.
Syncaris sp.
Tanyderidae
Tanypodiinae
Tholymis tillarga
Tipula sp.
Tomocerus sp.
Unio douglasiae
Whitmania pigra Whitman

Carabidae, Ceratopogoniidae, Crambidae, Dolichopodidae, Dytiscidae, Elmidae, Ephydridae, Hydrophilidae, Leuctuidae, Limnephilidae, Molannodes, Oreoleptidae and *Tanyderidae* were identified to family level; *Nematoda* and *Oligochaeta* were only identified to class level.

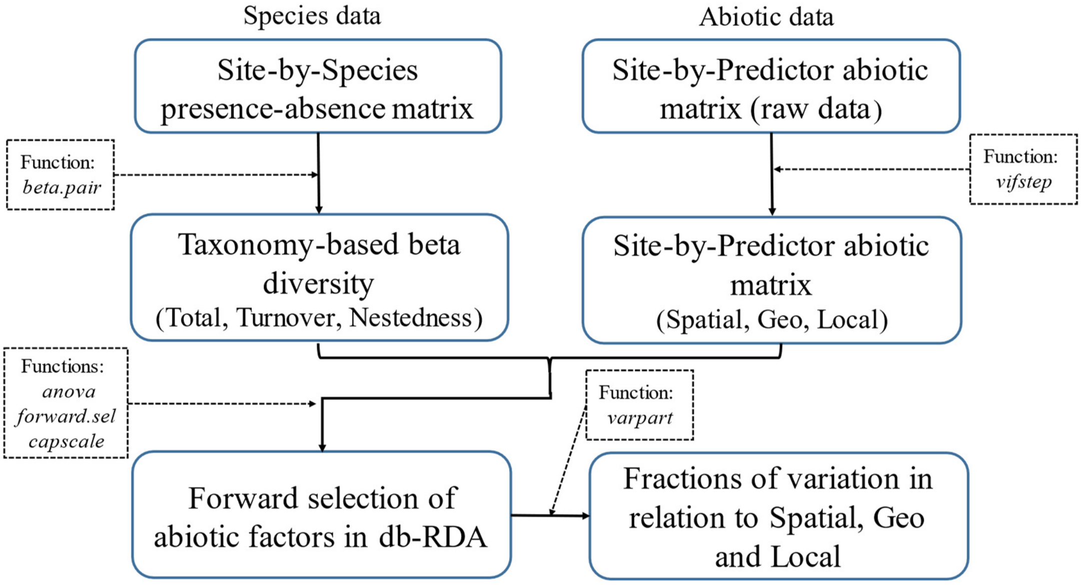

Figure S1. A flow-chart of taxonomic β-diversity analyses.

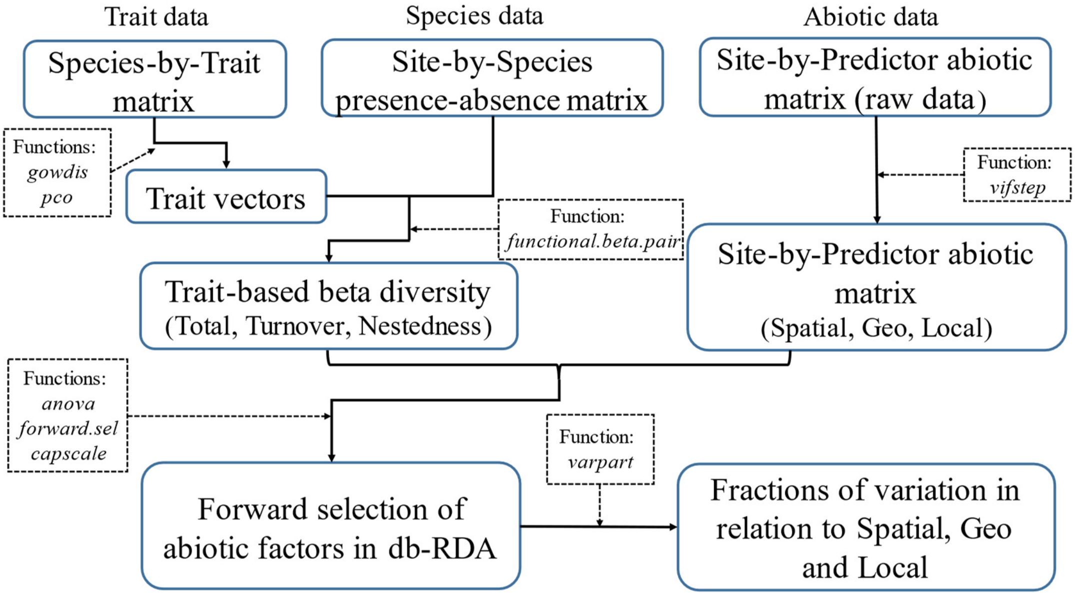

Figure S2. A flow-chart of functional *β*-diversity analyses.

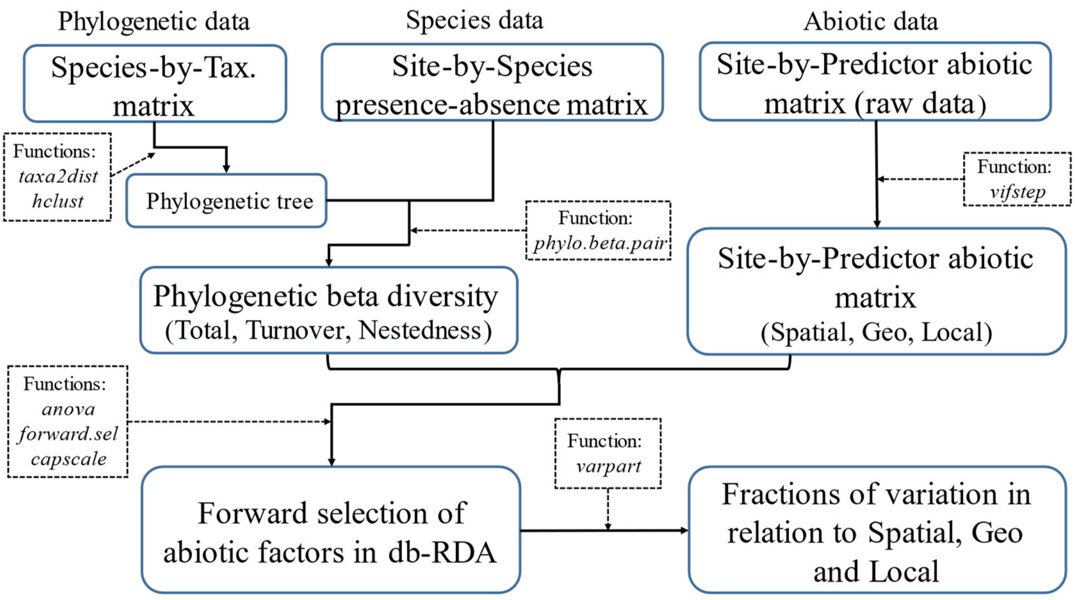

Figure S3. A flow-chart of phylogenetic β-diversity analyses.
